# The evolutionary advantage of heritable phenotypic heterogeneity

**DOI:** 10.1038/s41598-017-05214-2

**Published:** 2017-07-11

**Authors:** Oana Carja, Joshua B. Plotkin

**Affiliations:** Department of Biology, University of Pennsylvania, Philadelphia, 19104 USA

## Abstract

Phenotypic plasticity is an evolutionary driving force in diverse biological processes, including the adaptive immune system, the development of neoplasms, and the persistence of pathogens despite drug pressure. It is essential, therefore, to understand the evolutionary advantage of an allele that confers on cells the ability to express a range of phenotypes. Here, we study the fate of a new mutation that allows the expression of multiple phenotypic states, introduced into a finite population of individuals that can express only a single phenotype. We show that the advantage of such a mutation depends on the degree of phenotypic heritability between generations, called phenotypic memory. We analyze the fixation probability of the phenotypically plastic allele as a function of phenotypic memory, the variance of expressible phenotypes, the rate of environmental changes, and the population size. We find that the fate of a phenotypically plastic allele depends fundamentally on the environmental regime. In constant environments, plastic alleles are advantageous and their fixation probability increases with the degree of phenotypic memory. In periodically fluctuating environments, by contrast, there is an optimum phenotypic memory that maximizes the probability of the plastic allele’s fixation. This same optimum memory also maximizes geometric mean fitness, in steady state. We interpret these results in the context of previous studies in an infinite-population framework. We also discuss the implications of our results for the design of therapies that can overcome persistence and, indirectly, drug resistance.

## Introduction

Perpetual volatility in the surrounding microenvironment, nutrient availability, temperature, immune surveillance, antibiotics or other drugs are realities of life as a microorganism^1–3^. To persist in constantly changing environments and increase resilience, microbial populations often employ mechanisms that expand the range of phenotypes that can be expressed by a given genotype^3, 4^. This form of bet-hedging may not confer an immediate fitness benefit to any one individual, but it can sometimes act to increase the long-term survival and growth of an entire lineage^5, 6^.

Phenotypic heterogeneity has been well documented both in the context of cellular noise without any known environmental triggers^7^ and in the context of persistent environmental challenges^8^. Classic examples include the bifurcation of a genotypically monomorphic population into two phenotypically distinct bistable subpopulations^1^, or phase variation, a reversible switch between different phenotypic states driven by differences in gene expression^3, 4, 9^. In order to motivate our modeling study, we first describe and compare four clinically relevant examples of phenotypic bet-hedging.

One of the most striking examples of evolutionary bet-hedging is bacterial persistence^6, 10–12^, whereby a genetically monomorphic bacterial population survives periods of large antibiotic concentrations by producing phenotypically heterogeneous sub-populations, some of which are drug-insensitive^13^. Persister phenotypes constitute dormant, transient phenotypic (epi)states, protected from the action of drugs. Phenotypic variants that can survive treatment differ from traditional resistance mutants, because the dormant phenotypes do not rely on genetic mutations^13^, though the mechanisms that lead to persistence are still under debate. The dormant phenotypic state can be partially heritable upon cellular division, so that the offspring cell can “remember” and express the phenotypic state of its parent with a certain probability. In such bacterial populations, individuals typically acquire and relinquish the dormant phenotype at rates that exceed the rate of DNA mutation^14, 15^, providing populations with the phenotypic plasticity required to persist through periodic environmental stresses. Even though persisters are a non-genetic form of inheritance, the capacity to generate persistent cells, and the propensity to retain the phenotype of a parent, are likely under genetic control^16^.

There are surprising parallels between persistent phenotypes in bacteria and the quiescent phenotype in cancer cell populations^17^. Although genetic resistance alleles and their role in tumor dynamics have been a focus in cancer research, there has been less study of drug-tolerant epi-phenotypes and their impact on the evolution of a neoplasm^18, 19^. Under drug concentrations that can eradicate some cancer cells, these epi-phenotypes can preserve viability by producing genetically identical, but slower-dividing sub-populations. The slower-dividing cells are maintained in the population by their ability to revert to the faster-growing phenotype in the absence of drug pressure. The drug-tolerant epi-state can be acquired, inherited, and relinquished by cells at rates much higher than that of genetic mutation and, similar to bacterial persisters, constitutes a mechanism by which protected subpopulations of cells can escape periods of high drug concentrations. Just like bacterial persisters, this bet-hedging strategy confers on the population a degree of phenotypic heterogeneity that helps it withstand periods of environmental stress.

Neoplastic cell populations employ other bet-hedging strategies during growth and spread. Depending on mechanical cues in the tumor microenvironment, for example, populations of cancer cells can utilize heterogeneity in the fast- or slow-locomotion phenotype, producing radically different evolutionary dynamics of cancer cell populations^20^. Genetically identical cells can express and switch between different motility states. Changing the tumor microenvironmental cues alters the rate of switching between the slow- locomotion mode and the fast-locomotion mode, and therefore changes the number of cells expressing either motility phenotype^20^.

Environmental shifts also occur when pathogens are transmitted between hosts. Bet-hedging in this context can manifest as a form of life-history strategy^21, 22^. Viral latency is a heterogeneous fate decision that can help a viral population persist and adapt as it encounters different host immune environments. Periods of latency are intervals in which a pathogen is non-transmissible but from which it may emerge in transmissible form. Examples include the choice between HIV latency or active replication upon infecting a CD4+ T lymphocyte^21, 23, 24^, or latency in herpes viruses^25^. Other examples include *Helicobacter pylori*, which establishes lifelong infection characterized by long periods of minimal transmissibility punctuated by infectiousness; *Salmonella typhi*, which can establish a latent reservoir in the gallbladder, and *Mycobacterium tuberculosis*, infection with which is characterized by unpredictable intervals of clinical latency (weeks-decades long). Even parasitic infections, such as *Strongyloidiasis*, can be characterized by periods of latency punctuated by high transmissibility. A probabilistic switch that produces both infectious cells and latency with the possibility of later infectivity may confer a competitive advantage in a fluctuating environment, driven by differences in the numbers of susceptible hosts or variation in host immune system repertoires over time. The evolutionary advantage of such a phenotypic switch, and how it depends on environmental variability, is precisely the topic of this study.

The tradeoffs of phenotypic variability in viral latency, or neoplastic motility are similar to those for antibiotic persisters or quiescent cancer cells: decreased environmental uncertainty versus decreased average replication rates. There are striking similarities among the four examples presented above: all involve genetically identical populations, with two or more available phenotypes, with each phenotype beneficial in a different environmental state. Phenotypic states are partly heritable by offspring cells. And yet individuals can switch between phenotypic states at rates that greatly exceed those of genetic mutations. Finally, the rate of ‘phenotypic mutation’ is itself under genetic control. These types of epigenetic bet-hedging strategies by dynamic regulation of phenotypic variability may allow the persistence of a population until more permanent genetic strategies can be found^26, 27^. Although the idea of phenotypic plasticity, and even subsequent genetic assimilation, dates back to Waddington^28^, its critical role in microbial population dynamics has only recently been appreciated^29–31^.

The ubiquity of heritable phenotypic heterogeneity demands rigorous study of its evolutionary dynamics in a population-genetic framework. Here we study the evolutionary fate of an allele that permits the expression of multiple phenotypic states, in a finite population of fixed size under either constant or periodically fluctuating environments. We imagine these phenotypic states as partially heritable, so that an expressed phenotype will be inherited by the offspring with some probability, *p*. We call this probability *p* the phenotypic memory. We study the probability of fixation of a new mutation that permits this form of heritable, increased variance in the expressed phenotype, in a population that is otherwise composed of individuals that are unable to express variable phenotypes. We are especially interested in the fate of such a mutation as a function of the variance in expressible phenotypes that it confers, the extent of phenotypic memory between generations, the rate of environmental change, and the population size. We will show that, in constant environments, increasing phenotypic memory always increases the fixation probability of such a phenotypically-variable mutant. In contrast, in periodically changing environments there is an intermediate rate of phenotypic memory that maximizes the fixation probability of such a new mutant. Moreover, the phenotypic memory that maximizes fixation probability is proportional to the rate of environmental change, and it is largely insensitive to the population size or to the expressible phenotypic variance of the mutant type. Furthermore, we show that the phenotypic memory that maximizes invasion probability also maximizes geometric mean fitness in mutation-selection-drift stationary state, and we discuss these results in the context of prior studies in an infinite population.

We explore these theoretical questions about phenotypic heterogeneity with the eventual goal of making explicit mathematical predictions that inform treatment strategies. For example, the past few years have seen dramatic efforts in the search for epigenetic biomarkers and drivers of cancer, and concomitant development of epigenome-targeted therapies^32, 33^. These studies been focused on changes at the genome-wide CpG methylation level^34, 35^, which has been shown to be one of the mechanisms controlling phenotypic transitions^36^ and is hypothesized to play a key role in carcinogenesis^26^. The underlying motivation and implicit rational of epigenetic therapies has been to reverse the epigenetic modifications known to accrue during tumorigenesis. Nevertheless, fundamental questions, such as whether reversing epigenetic states is likely to be beneficial or likely to increase resistance, remain unanswered. More generally, in the context of diverse clinical problems where resistance can be indirectly achieved by transient, non-genetic cellular epistates, the question of how best to modify heritability of non-genetic phenotypes for a therapeutic outcome remains largely unstudied.

## Evolutionary bet-hedging models

### Previous theory

Phenotypic variability is often caused by switches between different regulatory states that produce bi- or multi-stability, due to fluctuations in levels of methylation at CpG sites, for example in mRNA transcription, or protein translation^37^. Our focus here, however, is not on the specific mechanisms or bio-physical forces that govern these phenotypic state transitions^9, 22, 38, 39^. Instead, we aim to elucidate the population-level consequences of increased phenotypic availability from a given genotype and how this non-genetic variability determines the long-term evolutionary outlook of a population^6, 40–42^. In this framework, the trade-offs between the evolutionary advantages of phenotypic variability and the costs of maladaptation is our principal object of inquiry. Mathematical models that incorporate stochastic fluctuations in selection pressure are already essential components of population-genetic theory^43–46^. Early works by Gillespie^47, 48^ established the geometric mean fitness principle, and showed that the evolution of a population under fluctuating selection is controlled not only by the mean fitness in a generation but also by the variance in fitness between generations, so that an allele with higher mean fitness can be outcompeted by one with lower mean fitness if the variance of the latter is sufficiently lower than of the former. In subsequent work, Gillespie also showed, for variation in fitness within a generation, that the strength of selection for reduced variance is inversely proportional to the population size^49–52^.

In all the models of variable fitness summarized above, the phenotype of an offspring is independent of the phenotype of its parent. The absence of phenotypic inheritance make these models a poor choice for studying the types of phenotypic heterogeneity described in our Introduction, which are often relevant in clinical settings. Heritability of phenotype is an essential requirement for evolutionary change to occur, and so (partial) heritability of an individual’s phenotype is an essential component of the models we develop here. The heritable phenotypic variation we study, which produces phenotypic variability with familial correlations, is an intermediate between genetically determined phenotypic variation, where offspring are phenotypically similar to parents, and phenotypic plasticity, which causes large phenotypic variation within a genetically clonal population but is not inherited by offspring.

Previous study of partly heritable phenotypic variation has been pursued in an infinite population^6, 40, 53, 54^, with the notable exception of work by ref. 55 studied bet-hedging against rare events, whose analysis involved considering a single environmental change. The evolutionary advantage of bet-hedging alleles depends on the rate of environmental change, as phenotypically variable alleles are often at a disadvantage during long periods of environmental stasis. The biological systems discussed in the introduction are all examples of frequent environmental change, where a bet-hedging allele can encounter multiple environmental changes while it segregates in the population. Thus, our analysis will focus on this regime. Our analysis will also account for the effects of genetic drift, neglected by infinite-population models. In particular, we study a problem that does not occur in an infinite population: the fixation probability of a new, plastic mutant and the strategies that maximize this fixation probability. This framework allows us to determine the dynamics of a novel persistence allele in the presence of demographic stochasticity.

### A population-genetic model

We use a Wright-Fisher-type model to describe changes in allele frequencies in a finite population of fixed size *N*. Each individual is defined by one biallelic locus *A*/*a*, which controls its phenotype. The *A* allele encodes a fixed phenotypic value, whereas individuals with the *a* allele may express a wider range of phenotypes: such an individual’s phenotype is drawn from a random variable with a fixed mean and variance. The phenotypic random variable associated with allele *a* can either be discrete (e.g. discrete epi-phenotypes around a fixed value), or continuous (e.g. a continuous phenotypic interval available to the persister genotype *a*). We consider a population initially fixed for the wild-type allele *A*, which expresses only a single phenotype. We introduce one copy of the *a* allele in the population and determine the fixation probability of this new mutation, which confers its holders with access to a larger, and partially heritable, phenotypic range.

**Figure 1:**
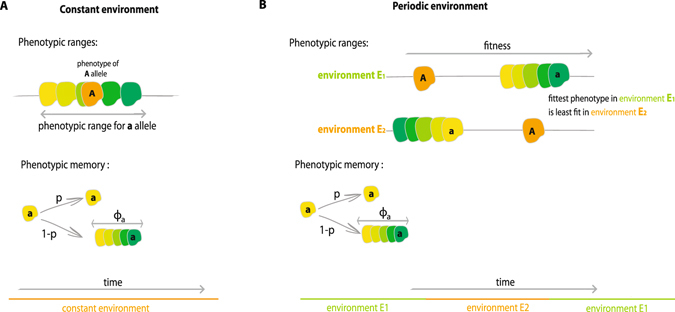
Illustration of model. (**A**) Constant environment. The population is initially fixed on allele *A*, which can express only one phenotype, Φ_*A*_. We determine the fate of a new mutant, *a*, which has access to a wider phenotypic range described by the random variable Φ_*a*_. Fitness is defined to equal phenotype, and both alleles have the same mean fitness: ${\mathbb{E}}({{\rm{\Phi }}}_{A})={\mathbb{E}}({{\rm{\Phi }}}_{a})$. For individuals of *a* genotype there exists a probability of phenotypic memory *p*, between parent and offspring: with probability *p*, the offspring keeps the same phenotypic state as its parent, while, with probability 1 − *p*, the offspring’s phenotype is determined as a new sample from Φ_*a*_. (**B**) Periodic environment. With environments changing, we assume that the phenotypes that are fitter in one environment are less fit in the other. Both alleles have the same mean fitness in their preferred environment: ${\mathbb{E}}({f}^{1}({{\rm{\Phi }}}_{A}))={\mathbb{E}}({f}^{2}({{\rm{\Phi }}}_{a}))$ and ${\mathbb{E}}({f}^{2}({{\rm{\Phi }}}_{A}))={\mathbb{E}}({f}^{1}({{\rm{\Phi }}}_{a}))$ and the phenotypic variance of the *a* allele is the same in both environments: Var (*f* ^1^(Φ_*a*_)) = Var (*f* ^2^(Φ_*a*_)) = Var (Φ_*a*_).

We study two versions of the model, one for a constant environment and one for a periodic environment (Fig. 1A,B). The mapping from phenotype to fitness depends on the environmental regime, as described below, and is chosen so that both alleles have the same expected fitness across environments. At each generation the population experiences selection and reproduction, followed by a possible phenotypic change for offspring of allelic type *a*. In particular, to create the next generation we choose *N* individuals to reproduce from the current population, sampled with replacement with probabilities proportional to their fitness relative to the population mean fitness. We then determine the phenotypic state of every offspring in the next generation as follows. If the individual chosen to reproduce has genotype *A*, then the phenotypic state of the offspring always equals its parent’s fixed phenotypic value. For the individual with the phenotypically-variable *a* allele, however, there exists a probability of phenotypic memory, denoted by the parameter *p*, between parent and offspring: with probability *p* the offspring retains the phenotypic state of its parent, and with probability 1 − *p* the offspring’s phenotype is drawn independently from the random variable Φ_*a*_. Thus, individuals of type *a* can express a range of phenotypic values, and their phenotype is partly heritable between generations (provided *p* > 0).

### Constant environment

The constant environment model studies the probability of fixation of a new allele that can express multiple phenotypic states, but has the same mean fitness as the wild-type. The fitness scheme takes the form

$$\begin{array}{lll}{\rm{Genotype}} & {\rm{A}} & {\rm{a}}\\ {\rm{Phenotype}} & {{\rm{\Phi }}}_{A} & {{\rm{\Phi }}}_{a}\\ {\rm{Fitness}} & {{\rm{\Phi }}}_{A} & {{\rm{\Phi }}}_{a}\end{array}$$

where Φ_*A*_ and Φ_*a*_ are random variables. The fitness of an individual is defined to be equal to its phenotype. The random variable Φ_*A*_ is in fact deterministic with variance zero–that is, an individual with the *A* allele can express only one phenotype. By contrast, Φ_*a*_ is not deterministic and it can be either a discrete or continuous random variable with positive variance. Φ_*A*_ and Φ_*a*_ are chosen so that two alleles have the same mean fitness: ${\mathbb{E}}({{\rm{\Phi }}}_{A})={\mathbb{E}}({{\rm{\Phi }}}_{a})$. We choose fitness functions with equal means so as to focus our analysis on the effect of variance in phenotypes expressed by allele *a*, Var (Φ_*a*_), and not on any mean-fitness effect. An illustration of this model is presented in Fig. 1, Panel A.

This model was first introduced by Gillespie^49, 50^, with the exception that, in previous studies, the expressed phenotypic states were not inherited between generations, but rather always redrawn from the phenotypic distribution. Thus Gillespie’s model corresponds to the “no-memory” case (*p* = 0) of the more general model we have defined. In the absence of memory, Gillespie showed that phenotypic variance Var (Φ_*a*_) can sometimes introduce fitter phenotypes into the population, but that it nonetheless carries an overall fitness cost for the *a* genotype. Here, we investigate whether these classic results still hold when phenotypes are partially heritable between generations.

### Periodic environment

In the periodic environment model, we assume that the population experiences two different environments, *E*_1_ and *E*_2_, which alternate deterministically every *n* generations, so that the both environments are experienced every 2*n* generations. We assume that one environment is more favorable on average to one allele, and the other environment to the other allele. We describe a scenario where the plastic allele *a* has, on average, lower fitness than the wild-type allele in one of the environmental regimes. For example, in the case of persister phenotypes, this would correspond to the “no antibiotic” environment. In the presence of antibiotic pressure, by contrast, the expected growth rate of the persister allele *a* is higher than the wild-type *A*.

Just as in the case of the constant environment model, we choose phenotypic ranges and fitness functions so that the mean fitness of phenotype expressed by each of the two genotypes are equal, for a randomly drawn time. This model allows us to focus on the evolutionary advantage of phenotypic variance of *a*, Var (Φ_*a*_), and to discuss its consequences for different therapy strategies, without conflating its effect with any mean-fitness advantage of the *a* allele over the *A* allele.

The fitness scheme under a periodic environment can be written as

$$\begin{array}{lll}{\rm{Genotype}} & {\rm{A}} & {\rm{a}}\\ {\rm{Phenotype}} & {{\rm{\Phi }}}_{A} & {{\rm{\Phi }}}_{a}\\ {\rm{Fitness}}\,{\rm{in}}\,{\rm{environment}}\,{E}_{1} & {f}^{1}({{\rm{\Phi }}}_{A}) & {f}^{1}({{\rm{\Phi }}}_{a})\\ {\rm{Fitness}}\,{\rm{in}}\,{\rm{environment}}\,{E}_{2} & {f}^{2}({{\rm{\Phi }}}_{A}) & {f}^{2}({{\rm{\Phi }}}_{a}),\end{array}$$

where Φ_*A*_ and Φ_*a*_ are random variables and the random variable Φ_*A*_ is, again, deterministic with zero variance. The functions ${f}^{i}:{\mathbb{R}}\to {\mathbb{R}}$ (*i* ∈ {1, 2}) map phenotype to fitness in each of the two environments, and *f* ^1^ is the identity function. We assume that both alleles have the same mean fitness in their preferred environment, and the same mean fitness in their unpreferred environment: ${\mathbb{E}}({f}^{1}({{\rm{\Phi }}}_{A}))={\mathbb{E}}({f}^{2}({{\rm{\Phi }}}_{a}))$ and ${\mathbb{E}}({f}^{2}({{\rm{\Phi }}}_{A}))={\mathbb{E}}({f}^{1}({{\rm{\Phi }}}_{a}))$. This condition also ensures that the average of two alleles’ mean fitnesses, which we denote $m=\frac{{\mathbb{E}}({f}^{1}({{\rm{\Phi }}}_{A}))+{\mathbb{E}}({f}^{1}({{\rm{\Phi }}}_{a}))}{2}=$ $\frac{{\mathbb{E}}({f}^{2}({{\rm{\Phi }}}_{A}))+{\mathbb{E}}({f}^{2}({{\rm{\Phi }}}_{a}))}{2}$, is the same in both environments. The function *f* ^2^ is defined as a reflection of *f* ^1^ around *m*: for any phenotype *x*, *f* ^2^(*x*) = 2*m* − *f* ^1^(*x*). As a result, the variance in fitness of allele *a* is the same in both environments: Var (*f* ^1^(Φ_*a*_)) = Var (*f* ^2^(Φ_*a*_)) = Var (Φ_*a*_). These fitness functions describe a model in which each genotype has one preferred environment, but allele *a* can express a range of phenotypes whereas allele *A* expresses only a single phenotype in each environment (see illustration in Fig. 1, Panel B). The symmetry conditions we have imposed on phenotypic means allow us to focus our analysis on the effects of phenotypic variation alone.

In both environmental regimes, we study the possible long-term advantage of the phenotypic plasticity by analyzing the fixation probability of a phenotypically variable *a* allele introduced into a population otherwise composed of the non-variable *A* allele. How does this probability of fixation depend on environmental factors, such as the environmental period 2*n*, on demographic factors, such as the population size *N*, and on molecular factors, such as the variance in phenotypes that can be expressed by *a*, Var (Φ_*a*_), or the degree of phenotypic memory, *p*?

Although part of our analysis is analytical, we primarily determine the probability of fixation in changing environments by Monte Carlo simulation. We estimate this probability by simulating an ensemble of 10,000 replicate populations. An ensemble this large allows us to reject the null hypothesis of a neutral fixation probability with power 0.8, and to distinguish between fixation probabilities as similar as 0.001 and 0.002. We initiate all populations with one copy of the *a* allele, and we simulate the process until fixation of one of the two alleles, or discard simulations that have not absorbed after 10,000 generations (a conservative cutoff, leading to extremely few discards see Supplementary Figure S1).

## Results

### Constant environment results

In a constant environment, previous studies found that phenotypic variability within a generation is associated with an overall fitness cost to that plastic allele^49, 50^. We recapture these results in the case of no phenotypic memory (*p* = 0). However, the size of this fitness cost is extremely small in general, and in fact, so small in the regimes we study, that the fixation probability of the *a* allele is not statistically different from 1/*N*, with power 0.8.

**Figure 2:**
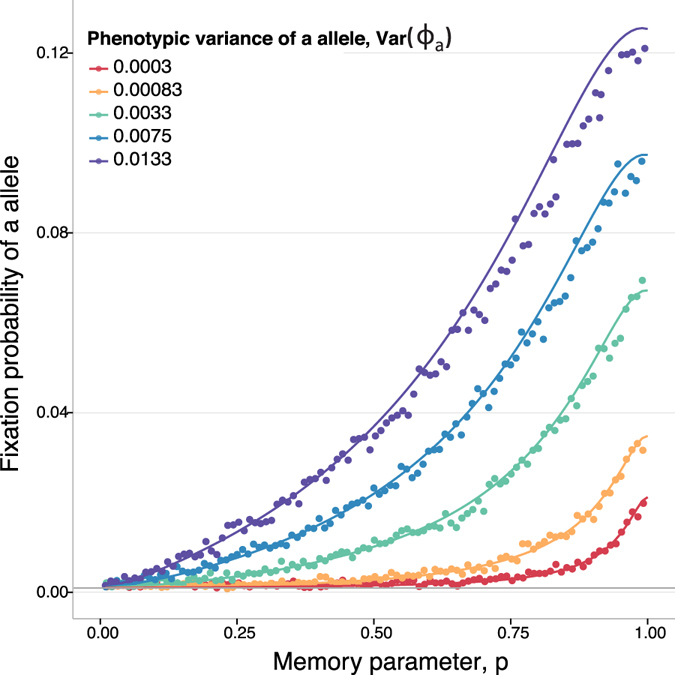
Fixation probability of a phenotypically variable allele in a constant environment. The phenotype of the *A* allele is fixed at Φ_*A*_ = *δ*_0.8_. The *a* allele only has access to two equiprobable discrete phenotypes, one with higher fitness than the *A* allele, and one with lower fitness, with mean also equal to 0.8. Colors represent different phenotypic variances, with Var (Φ_*a*_) presented in the legend. The dots show the fixation probability of a new *a* allele, introduced with equal probability of either phenotype, into a population of size *N* = 1000 with resident *A* allele. The fixation probability, determined by Monte Carlo simulation, is shown as a function of the phenotypic memory parameter *p*. The grey line is drawn at 1/*N*. The curves represent analytical approximations.

What happens to the classic result on the cost of phenotypic variance when we allow phenotypes to be inherited between generations? We find that, for *p* > 0, the classical result no longer holds, and the plastic allele enjoys a selective advantage compared to the wild-type *A* allele. Moreover, this fixation probability is an increasing function of phenotypic memory, *p* (Fig. 2). For larger values of phenotypic memory, the selective advantage of the phenotypically heterogeneous allele is increased, and this increase is more pronounced when the *a* allele can express a greater diversity of phenotypes, i.e. for Var (Φ_*a*_) large. There is a simple intuition to explain these results. The high-fitness variants of the *a* allele are preferentially transmitted to the next generation, giving the *a*-lineage an advantage; and the phenotypic memory *p* gives such individuals the opportunity to remain high-fitness instead of being resampled from the whole distribution Φ_*a*_. As a result, the mean fitness within the *a*-allele subpopulation is increased by phenotypic memory, while the variance within the *a* subpopulation is decreased. According to this simple intuition the fixation probability of the phenotypically variable *a* allele will always be greater when *a* alleles can express a broader range of phenotypes, that is larger Var (Φ_*a*_), as confirmed in Fig. 2.

Our result on the advantage of phenotypic memory depends on whether the phenotypic memory is individual-based. In other words, in our model, each offspring of an *a*-type parent independently chooses whether to retain its parent’s phenotype, and, if not, draws a new phenotype independently from all other offspring. Under an alternative model that assumes “global” choices for inheritance of phenotypes, phenotypic memory has a much smaller effect on the evolutionary dynamics: the plastic allele has an evolutionary advantage only for very large values of memory (Supplementary Figure S2). Under that form of global sampling, all *a*-type offspring inherit their parental phenotype with probability *p*, or they simultaneously redraw the same new phenotype with probability (1 − *p*). Thus, under global phenotypic memory, all individuals of allelic type *a* express the same phenotype in each generation, similar to a model of between-generation phenotypic variance studied by Gillespie^47^. This form of global phenotypic resampling does not confer the same advantage to high-fitness phenotypic realizations of the *a* allele, because low-fitness individuals are just as likely to express and retain their phenotype as high-fitness individuals (Supplementary Figure S2). And so, in summary, phenotypic memory provides an significant advantage to a plastic allele *a* only when individuals independently inherit or re-sample phenotypes, as is the case for epi-genetic mechanisms of phenotypic change.

Figure 2 shows the results of Monte Carlo simulations together with an analytic approximation for the probability of fixation of the plastic *a* allele, in the case of a discrete random variable Φ_*a*_ with two available phenotypes, Φ_*a*,*min*_ and Φ_*a*,*max*_ (as illustrated in Supplementary Figure S3). The probability of fixation of the *a* allele is invariant to the choice of phenotypic distribution, continuous or discrete, provided the distributions have equal variance (Supplementary Figure S4, Panels A, B and C).

To derive an analytical understanding of fixation rates, we first derive the probability of fixation under the assumption that the *a* mutation is introduced with phenotype Φ_*a*,*max*_, ${{\mathbb{P}}}_{f}({a}_{max})$. To do so, we compute an effective selection coefficient for the entire, phenotypically variable *a* lineage, by assuming that the two phenotypes within the *a* lineage quickly reach mutation-selection balance. Given “mutation” rate $\mu =\frac{1-p}{2}$ between the two phenotypes, at equilibrium, the frequency of phenotype Φ_*a*,*max*_ within the *a*-lineage is given by *f*_*a*,*max*_:

1 $$\begin{array}{rcl}{f}_{a,max} & = & \frac{{{\rm{\Phi }}}_{a,max}-{{\rm{\Phi }}}_{a,min}-\mu {{\rm{\Phi }}}_{a,min}-\mu {{\rm{\Phi }}}_{a,max}}{\mathrm{2(}{{\rm{\Phi }}}_{a,max}-{{\rm{\Phi }}}_{a,min})}\\  &  & +\frac{\sqrt{4{{\rm{\Phi }}}_{a,min}\mu ({{\rm{\Phi }}}_{a,max}-{{\rm{\Phi }}}_{a,min})+{({{\rm{\Phi }}}_{a,min}-{{\rm{\Phi }}}_{a,max}+\mu {{\rm{\Phi }}}_{a,max}+\mu {{\rm{\Phi }}}_{a,min})}^{2}}}{\mathrm{2(}{{\rm{\Phi }}}_{a,max}-{{\rm{\Phi }}}_{a,min})}\end{array}$$

We can alternatively express *f*_*a*,*max*_ as a function of the phenotypic mean and variance of the *a* allele, to provide intuitive understanding of how the probability of fixation scales with other parameters of the model:

2 $${f}_{a,max}=\frac{2{\sigma }_{{{\rm{\Phi }}}_{a}}-2{\mathbb{E}}({{\rm{\Phi }}}_{a})\mu +\sqrt{{\mathrm{(2}{\mathbb{E}}({{\rm{\Phi }}}_{a})\mu -2{\sigma }_{{{\rm{\Phi }}}_{a}})}^{2}-8{\sigma }_{{{\rm{\Phi }}}_{a}}({\sigma }_{{{\rm{\Phi }}}_{a}}\mu -{\mathbb{E}}({{\rm{\Phi }}}_{a})\mu )}}{4{\sigma }_{{{\rm{\Phi }}}_{a}}}\mathrm{.}$$

We then compute the effective selective coefficient of the *a* lineage compared to the *A* lineage as

3 $${s}_{a}=\frac{{{\rm{\Phi }}}_{a,min}\mathrm{(1}-{f}_{a,max})+{{\rm{\Phi }}}_{a,max}\,{f}_{a,max}}{{{\rm{\Phi }}}_{A}}-\mathrm{1,}$$

or, alternatively, as a function of ${\mathbb{E}}({{\rm{\Phi }}}_{a})$ and ${\sigma }_{{{\rm{\Phi }}}_{a}}$, as

4 $${s}_{a}=\frac{({\mathbb{E}}({{\rm{\Phi }}}_{a})-{\sigma }_{{{\rm{\Phi }}}_{a}}\mathrm{)(1}-{f}_{a,max})+({\mathbb{E}}({{\rm{\Phi }}}_{a})+{\sigma }_{{{\rm{\Phi }}}_{a}}){f}_{a,max}}{{\mathbb{E}}({{\rm{\Phi }}}_{a})}-1.$$

Finally, using the classic result of Kimura^56, 57^, the fixation probability of such an *a* allele can be approximated by ${{\mathbb{P}}}_{f}({a}_{max})=\frac{1-{e}^{-2{s}_{a}}}{1-{e}^{-2N{s}_{a}}}$.

This mathematical formalism allows us to observe that the population size *N* leads to differences in the fixation probability only when memory *p* is very small and the population dynamics approach neutrality, otherwise its contribution to ${{\mathbb{P}}}_{f}({a}_{max})$ is vanishingly small.

Conversely, when the *a* mutation is introduced with phenotype Φ_*a*,*min*_, we can derive an approximation for ${{\mathbb{P}}}_{f}({a}_{min})$ as the probability of at least one phenotypic mutation to Φ_*a*,*max*_ before the loss of the allele, multiplied by ${{\mathbb{P}}}_{f}({a}_{max})$. We denote the count of allele *a* in generation *t* by *X*_*t*_. Let *η* denote the event that there is at least one phenotypic mutation within the *a*-lineage before its loss. The frequency of a deleterious allele with selection coefficient −*s*, introduced in one copy, is expected to decay e^−*st*^, and so

5 $${\mathbb{P}}(\eta )=1-\prod _{t=0}^{\infty }{\mathrm{(1}-\mu )}^{{X}_{t}}=1-{\mathrm{(1}-\mu )}^{{\sum }_{t=0}^{\infty }{X}_{t}},$$

where

6 $$\sum _{t=0}^{\infty }{X}_{t}=\sum _{t=0}^{\infty }{{\rm{e}}}^{-st}=\frac{1}{1-{{\rm{e}}}^{-s}}.$$

Finally, the fixation probability of a novel allele *a* with uniformly random initial phenotype is then:

7 $$\begin{array}{rcl}{{\mathbb{P}}}_{f}(a) & = & \frac{1}{2}{{\mathbb{P}}}_{f}({a}_{max})+\frac{1}{2}{{\mathbb{P}}}_{f}({a}_{min})=\frac{1}{2}{{\mathbb{P}}}_{f}({a}_{max})+\frac{1}{2}{\mathbb{P}}(\eta ){{\mathbb{P}}}_{f}({a}_{max})\\  & = & {{\mathbb{P}}}_{f}(a)=\frac{1}{2}(2-{\mathrm{(1}-\mu )}^{\frac{1}{1-{{\rm{e}}}^{-s}}}){{\mathbb{P}}}_{f}({a}_{max}\mathrm{).}\end{array}$$

This approximation is shown together with the Monte Carlo simulations in Fig. 2.

### Periodic environment results

The role of phenotypic memory is fundamentally different in a periodic environment as opposed to a constant environment, even though we choose fitness functions so that both alleles have the same expected fitness, across environmental regimes. Whereas in a constant environment greater phenotypic memory always increases the advantage of the phenotypically variable *a* allele, we find qualitatively different behavior in a periodically changing environment: the fixation probability of the *a* allele is no longer monotonically increasing with phenotypic memory, but rather there exists an intermediate memory p* that maximizes the fixation probability.

**Figure 3:**
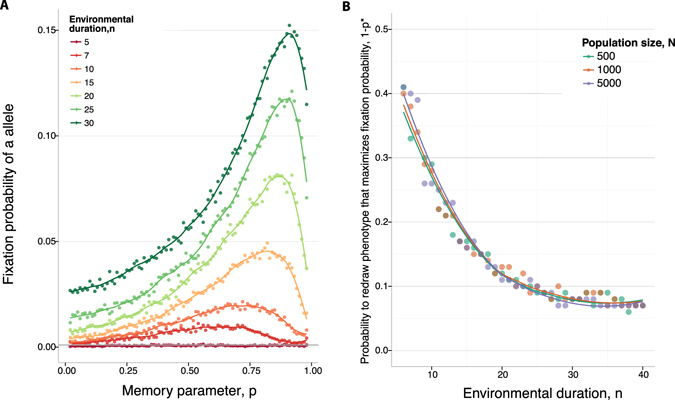
Fixation of a phenotypically variable allele in a periodic environment. (**A**) The dots show the fixation probability of a novel *a* allele in a population of size *N* = 1000 as a function of the phenotypic memory parameter *p*, determined by Monte Carlo simulation. In all cases we set *f* ^1^(Φ_*A*_)) = 0.6 and *f* ^2^(Φ_*A*_)) = 0.8. The fitness of an *a* allele in the first environment, *f* ^1^(Φ_*a*_) is sampled uniformly in the interval [0.7, 0.9], wheres *f* ^2^(Φ_*a*_) is uniform on the interval [0.5, 0.7]; and so that each allele as the same expected fitness in its preferred environment. The initial environment is chosen at random. The colors represent different rates of environmental change, as presented in the legend. (**B**) The environmental duration is plotted on the *x*-axis against 1 − p*, where p* is the value of phenotypic memory that maximizes the probability of fixation of a novel *a* allele. Different colors represent different population sizes, *N*, as indicated. The phenotypic variance of the *a* allele, Var (Φ_*a*_), is set to 0.0033. The curves show cubic spline fits to the simulated data.

The fixation probability of the plastic *a* allele is shown in Fig. 3, Panel A as a function of the strength of phenotypic memory *p*, for a random starting environment. The non-monotonicity we observe makes intuitive sense. On the one hand, it is beneficial to the *a* allele to have at least some phenotypic memory within each environment (*E*_1_ or *E*_2_), for the reasons described above in the case of a constant environment. But on the other hand, too much phenotypic memory is deleterious in a periodically changing environmental regime, because once the environment shifts, the *a* lineage would benefit from changing its phenotype to the new optimum. According to this simple intuition, the optimal phenotypic memory p* should be smaller when the environment fluctuates more rapidly, as can be seen in Fig. 3, Panel A (comparing, say, environmental duration *n* = 5 generations to *n* = 30 generations). Therefore, the maximum phenotypic memory p* beyond which further memory is deleterious, should scale with the environmental duration, as confirmed in Fig. 3, Panel B. The same figure shows that the value of p* that maximizes the fixation probably of the *a* allele is insensitive to variation in the population size *N*, at least over the order of magnitude variation that we investigated. The average time to fixation is presented in Supplementary Figure S5.

We also computed the counter-fixation probability of one *A* allele introduced in a population otherwise fixed for the *a* allele. The state of the population at the time of *A*’s introduction was drawn from the stationary distribution for the *a* phenotypes, as determined by burn-in simulations (Supplementary Figure S6). In the constant environment case (Panel A), the *A* allele invades with probability on the order of 1/*N* or lower. In the changing environment case (Panel B), the counter-invasion probability of *A* can exceed the neutral fixation probability for small memory, and it increases with the environmental period. This is strongly driven by the initial conditions: the longer the initial environment during which the A allele is preferred, the larger its ultimate fixation probability. Nonetheless, the counter-fixation rate of *A* is never larger than the forward fixation rate of *a*–and indeed, in the regimes of intermediate phenotypic memory, the counter-fixation probability of *A* is many orders of magnitude smaller than the fixation probability of *a*.

**Figure 4:**
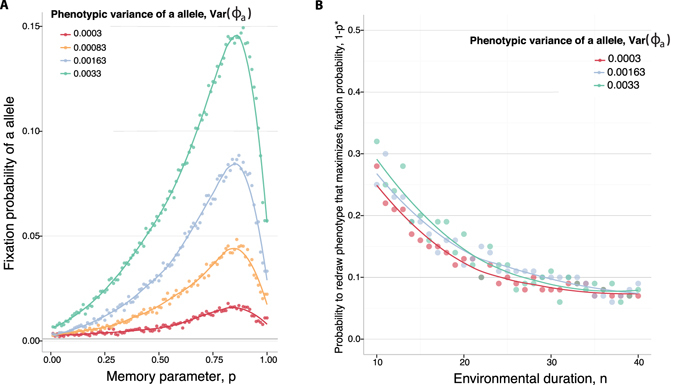
Fixation of a phenotypically variable allele in a periodic environment. (**A**) The phenotypic memory *p*, on the *x*-axis, is plotted against the fixation probability of a novel *a* allele in a population of size *N* = 1000. Different colors represent different variances of the *a* phenotype, Var (Φ_*a*_), as indicated. The duration of one environmental period is *n* = 20. The first environment is always *E*_1_, in this figure. (**B**) The environmental duration is plotted on the *x*-axis, against 1 − p*, where p* is the value of phenotypic memory that maximizes the probability of fixation in a population of size *N* = 1000. Different colors represent different phenotypic variances of the *a* allele, Var (Φ_*a*_), as indicated. The curves show cubic spline fits to the simulated data.

Regardless of the strength of phenotypic memory, the fixation probability of a new *a* allele always increases with the variance in phenotypes it can express, Var (Φ_*a*_) (Fig. 4, Panel A). Nonetheless, the optimum phenotypic memory for invasion, p*, is insensitive to the phenotypic variance of the *a* allele (Fig. 4, Panel B).

## Discussion

All cell populations harbor phenotypic variation, even cells that are genetically identical and in carefully controlled laboratory environments^7^. This stochastic variation is often regarded as merely experimental noise, and measurements are often averages of this variation^58^. However, transient and variable phenotypes can be mediated by transitions in the epigenome, and they may provide an additional, selectable layer of traits which is increasingly recognized as an evolutionary driving force in many biological systems. From bacterial infections, to the adaptive immune system, or neoplastic development, this form of phenotypic variability can provide a population with increased opportunity to adapt under varying selection pressures^5, 52, 59^.

We have studied the fate of an allele that endows the potential to express different, partly heritable phenotypes, in a population that otherwise lacks the capacity for phenotypic heterogeneity. We showed that the fixation probability of such an allele depends critically on the probability of phenotypic inheritance between parent and offspring, in both constant and fluctuating environments. Whereas in constant environments phenotypic memory always increases the fixation probability of a plastic allele, in periodically changing environments, by contrast, there exists an intermediate phenotypic memory that maximizes the invasion probability of the phenotypically plastic allele. In the clinical contexts of infection or tumor spread these results have an immediate qualitative implication: therapeutic interventions that either greatly reduce or greatly increase the heritability of cellular phenotypes/epi-states are expected to reduce the advantage otherwise enjoyed by plastic populations of pathogenic cells (e.g. persister bacterial cells or neoplastic subpopulations that vary in motility).

Previous theoretical studies on the adaptive benefit of phenotypic variability and bet-hedging have mostly considered the evolution of (epi) mutation rates in fluctuating environments by modifier loci, in an infinite population. These studies have found that (epi) mutation rates should evolve in tune with the correlation between the environments of parent and offspring^40, 53^. When the environment fluctuates periodically between two states with different optimal phenotypes, the uninvadvable switching rate between phenotypic states will evolve to approximately 1/*n*, where *n* is the number of generations between environmental changes. This uninvadable switching rate is an evolutionary stable strategy (ESS) in an infinite population^60^. Further studies confirmed these results and also generalized them to include both environmental and spatial fluctuations in selection^40, 54, 61^. Most of these studies, however, consider the dynamics of mutation rates in infinite populations, and they do not explore the evolutionary fate of a new mutation in a finite population subject to demographic stochasticity.

The problem of stochastic switching in finite populations has previously been studied by ref. 55, whose approach was later generalized by ref. 62. In both these studies, environmental changes were assumed to be vanishingly rare. By contrast, our study analyzes the problem of phenotypic plasticity when environmental changes are common. In particular^62^, studied evolution in the limit as rates of phenotypic switching and environmental changes both approach zero, which allowed them to use perturbation techniques. The evolutionary benefits of bet-hedging critically depend on the frequency of the environmental change. Previously^55^, had studied the evolutionary dynamics of phenotypic switching under the assumption that one environment is absorbing. Bet-hedging against these vanishingly rare events may require long periods of time since there is no immediate selection against lineages who do not hedge. In our work, by contrast to these prior studies, we have studied the fate of plastic alleles when environmental changes are common, such that the environment fluctuates repeatedly while the plastic allele is segregating within a population (Supplementary Figure S5). This regime is relevant to clinical settings such as persister phenotypes in microbial infections and epigenetic plasticity in tumor growth since these systems exhibit frequent environmental change that can impose strong selection for lineages able to express phenotypic variance.

The traditional definition of an ESS in an infinite population has little utility in finite populations, where even inferior alleles have a positive probability of fixation. As a result, the notion of strict ESS is typically inaccurate for predicting long-term evolutionary dynamics in finite populations^63, 64^. In an infinite population, the long term dynamics of a new mutant do not depend on the initial environment or phenotypes present in the population, whereas these factors can prove crucial for the early dynamics in a finite population. Fixation probabilities, on the other hand, are better metrics that can capture the long-term evolutionary dynamics in finite populations^64, 65^.

We have studied a problem that is similar to the one underlying the ESS–namely, finding the strategies that maximize the fixation probability of a new mutant in a finite population. We have found that these strategies behave similarly to the ESS strategies in infinite populations. This type of concordance between infinite and finite population models is relatively rare in population-genetic settings. Therefore, to further compare our results with those found in infinite populations we have also analyzed the case of a two-phenotype switching model (as illustrated in Supplementary Figure S7). Once again, we find an intermediate switching rate that maximizes the fixation probability of a new mutant in a finite population (Supplementary Figure S8). We have compared this optimum switching rate with the optimum ESS strategy previously derived in infinite populations^66^. Supplementary Figure S9 shows that these two optima, found in these two very different frameworks, are remarkably concordant, across a full order of magnitude in population size variation, extensive variation in environmental period, and also variation in phenotypic variance (not shown). We also compare results between systems characterized by different strengths of selection, for both the continuous and discrete switching models (Supplementary Figure S9), and show that these results also hold under a weaker selection regime.

**Figure 5:**
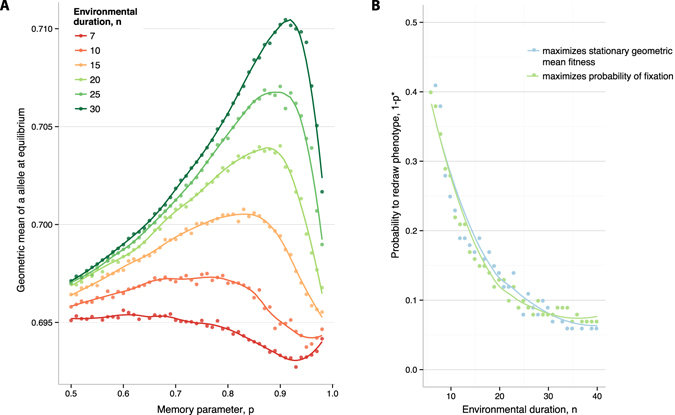
Fixation probabilty and geometric mean fitness. (**A**) Geometric mean fitness in stationary state, as a function of phenotypic memory *p*. The colors represent different environmental durations *n*, as indicated. Population size *N* = 1000. Variance of the *a* phenotype, Var (Φ_*a*_) = 0.0033. (**B**) Comparison of the phenotypic memory that maximizes the fixation probability and the memory that maximizes stationary geometric mean fitness. Population size *N* = 1000. Variance of the *a* phenotype, Var (Φ_*a*_) = 0.0033. The curves show cubic spline fits to the simulated data.

The two-phenotype models have a similar qualitative behavior as the multi-phenotype model described in the Results section. Nevertheless, it is important to note that the optimal switching rates in the case when the bet-hedging allele *a* has access to multiple phenotypic states is consistently higher than in the case of a switching *a* allele (compare, for example, Figs 3 and 5 with Supplementary Figure S8). This observation makes intuitive sense: when a continuum of phenotypic states is available, the selective consequences of a phenotypic change are typically less drastic than in the case of a switch to an automatically deleterious (or beneficial) phenotypic state, and so selection against a bet-hedging allele is weaker. This provides an extra dimension to the problem of the evolutionary advantage of bet-hedging, as incorporating multiple phenotypic states can lead to quantitatively different answers.

It is also useful to compare our finite-population analysis to results on ESS switching rates in terms of long-term geometric mean fitness^53^. Showed that, in infinite populations, the ESS switching rate maximizes a population’s long-term geometric mean fitness. This result has been generalized in related models of phenotypic switching, also in infinite populations^66^. To compare to these infinite-population results, we analyzed the evolutionary dynamics of a finite population fixed for the *a* allele, and we computed the geometric mean fitness across a full environmental period (that is, across 2*n* generations) of the mean phenotype in the population at each generation, after reaching stationary state distribution of phenotypes for *a* (determined using a burn-in simulation). We found that this stationary geometric mean fitness is, again, a non-monotonic function of the phenotypic memory parameter *p* (Fig. 5, Panel A). Moreover, the memory p* that maximizes the stationary geometric mean fitness also maximizes the fixation probability of a novel *a* mutant in the population (Fig. 5, Panel B). The coincidence of the phenotypic memory that maximizes fixation probability and the memory that maximizes stationary geometric mean fitness helps to explain the strong concordance between our finite-population results and the prior literature in infinite populations, where the relationship between ESS and geometric mean fitness was already established^53, 66^.

The goal of this study has been to develop a population-genetic model for the effects of bet-hedging in populations that harbor (partly) heritable variation in expressed phenotypes, subject to frequent environmental fluctuations. Although this study has been largely theoretical, neglecting the myriad mechanisms of phenotypic plasticity that arise in, and differ among, biological systems, the model we have developed nonetheless provides a logical basis for qualitative and, eventually, even quantitative predictions in biological systems. Such predictions are increasingly understood as required for rational design of therapies in clinical contexts, where phenotypic heterogeneity and epi-states inducing quiescent cellular states may be the primary driver of persistence and therapy failure across a diverse array of diseases^11, 67^. There is a need to consider the selective pressures on alleles that endow cellular populations phenotypic plasticity^19^. Our results provide insight into how, and under what conditions, such phenotypic heterogeneity can provide an initial selective advantage in changing environmental regimes.

Our results have immediate qualitative implications for therapy regimes already in common use for bacterial infections, namely antibiotic pulse therapies^68^. Figure 3 shows when a plasticity allele, which could allow microbes to persist during antibiotic treatment, is more likely to fix as the duration of environmental period increases. This theoretical result may help explain the observations in an experimental study by ref. 69. This study found that increasing pulse period of chloramphenicol treatment always increased the long-term growth rate of *E. coli*, however a different result is observed for a different type of antibiotic treatment, with bacterial growth maximized for intermediate period lengths. While that experimental study did not explicitly track persisters, it is possible that a significant proportion of the bacteria had the persister phenotype. Moreover, the stark discrepencies in bacterial growth between the different treatment options could be due to induced differences in phenotypic switching rates of these persisters.

To our knowledge no study has been able to directly measure the rates of phenotypic switching under a similar experimental setup. Here we theoretically explore the interplay between the rates of phenotypic switching and treatment period in driving bacterial growth. In this context, our results suggest that two very different types of intervention that will be effective. Both intervention options are based on the fact that, unlike genetic changes, such as mutations and chromosomal rearrangements, expression or phenotypic changes are reversible. The existence of an intermediate phenotypic heritability that maximizes the fixation probability of the plastic allele suggests an effective intervention by treatment with drugs that disrupt the molecular memory to either extreme (*p* = 0 or *p* = 1). Of course, further predictive models based on specific pathways, and unique to particular populations, are needed to formulate optimal therapies that minimize the spread of phenotypically-heterogeneous genotypes and resistance. And the most efficacious ways to implement these strategies remain to be defined.

## Electronic supplementary material

Supplementary Figures
