# Supplementary material for: The evolutionary advantage of heritable phenotypic heterogeneity
Source: Sci Rep. 2017 Jul 11;7:5090. doi: 10.1038/s41598-017-05214-2 (PMC5505965; doi:10.1038/s41598-017-05214-2)
Supplement: Supplementary file 1 — Supplementary Figures [file 41598_2017_5214_MOESM1_ESM.pdf]

Supplementary Information: The evolutionary advantage of heritable phenotypic heterogeneity

Oana Carja*[,1] and Joshua B. Plotkin[1]

[1]Department of Biology, University of Pennsylvania, Philadelphia, 19104.

* Correspondence to oana.carja@gmail.com

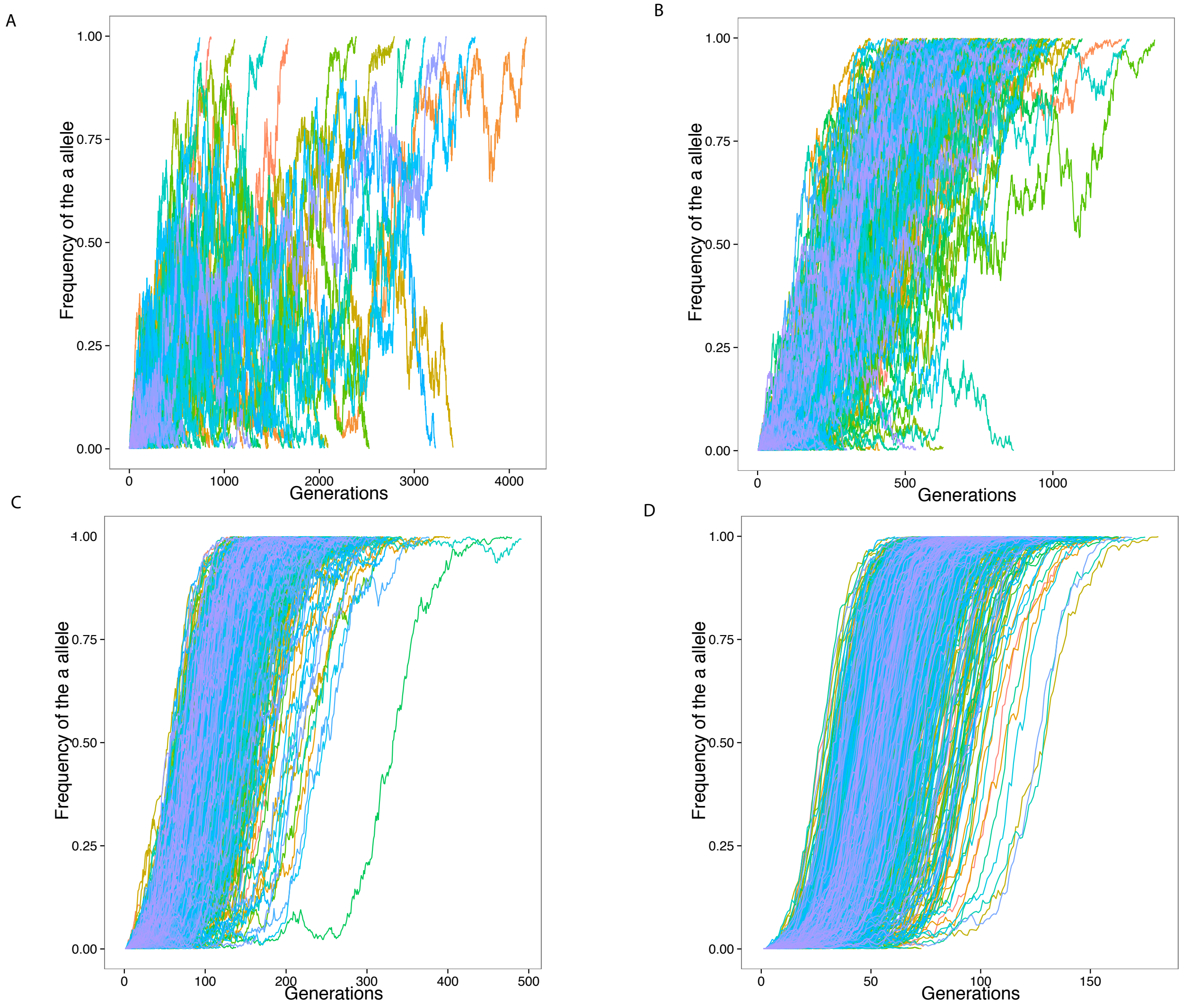

Figure S1: **Example sample paths for 10,000 runs of the simulation in a constant environment.** The phenotype of A allele fixed at $\Phi_A = \delta_{0.8}$. Mean phenotype of a allele is also 0.8, $\mathbb{E}(\Phi_a) = \delta_{0.8}$, with variance $\mathrm{Var}(\Phi_a) = 0.0133$. The y-axis shows the frequency of the a allele in the population. Population size $N = 1000$. **Panel A:** Memory parameter $p = 0$. **Panel B:** Memory parameter $p = 0.3$. **Panel C:** Memory parameter $p = 0.7$. **Panel D:** Memory parameter $p = 0.9$.

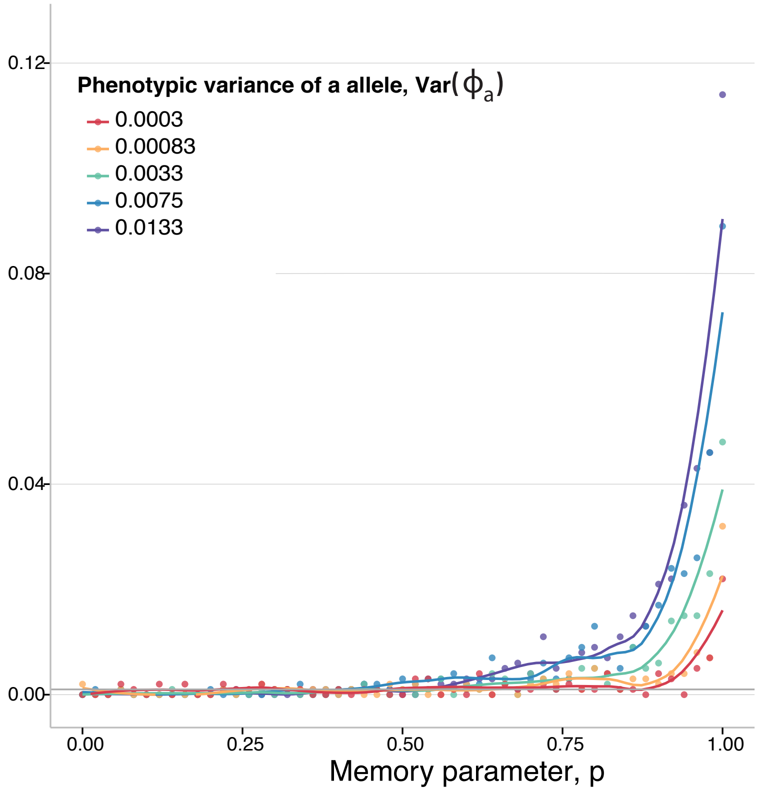

Figure S2: **The impact of global versus individual-specific memory in a constant environment.** A model where, every generation, all the individuals of a genotype draw the same phenotype. Moreover, at each generation, the probability of phenotypic memory p is globally determined for all individuals of a genotype. The phenotype of the A allele is fixed at $\Phi_A = \delta_{0.8}$. The distribution of phenotypes Φ_a that can be expressed by the a allele is assumed uniform with mean also equal to 0.8. Colors represent different phenotypic variances, with $\mathrm{Var}(\Phi_a)$ presented in the legend. Other parameters as in **Figure2**. The curves represent a fit to the data using a generalized additive model with penalized cubic regression splines.

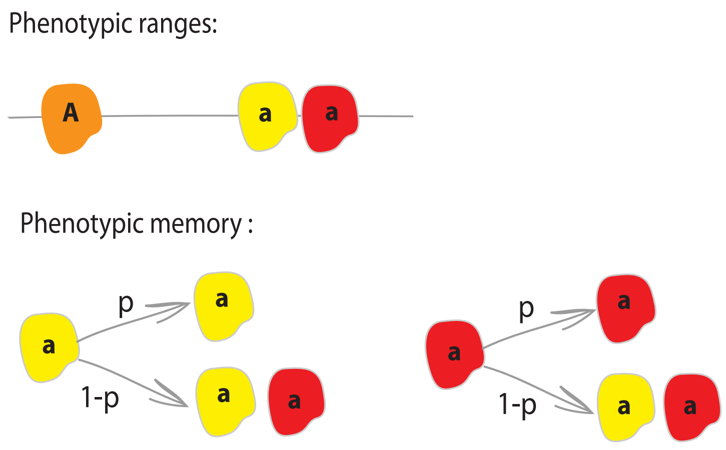

Figure S3: **Illustration of the constant environment model with discrete distribution.** The major differentiator of this simplified model is the fact that the a allele only has access to two discrete phenotypes, one with higher fitness than the A allele, and one with lower fitness.

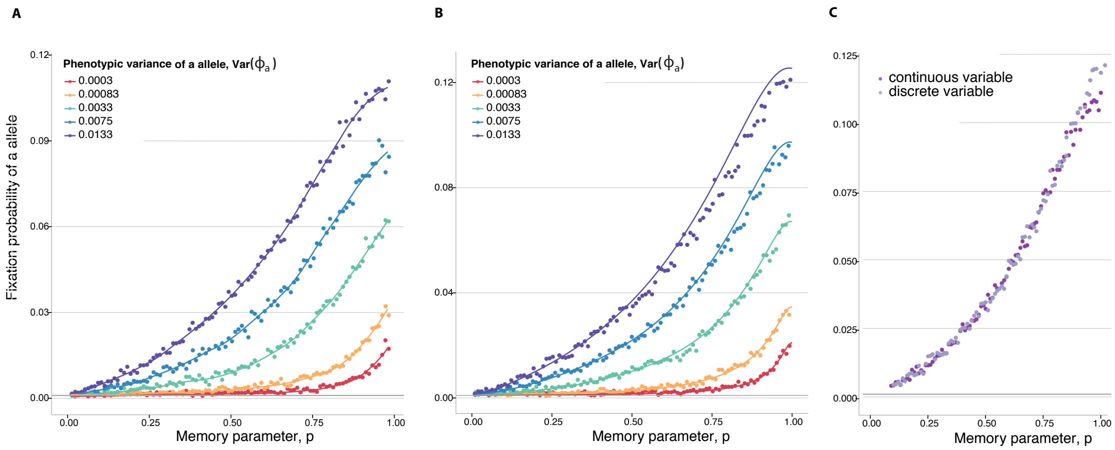

Figure S4: **Invasion probabilities in a constant environment. Panel A**: Continuous uniform phenotypic distribution of the a allele, equivalent to **Figure 2**. The curves represent a fit to the data using a generalized additive model with penalized cubic regression splines. **Panel B**: The discrete phenotypic distribution for a, as illustrated in **Figure S3** and presented in **Figure 2**. The curves represent the analytical approximation. **Panel C**: Comparison of simulation results between the continuous and the discrete models for $\mathrm{Var}(\Phi_a) = 0.0133$.

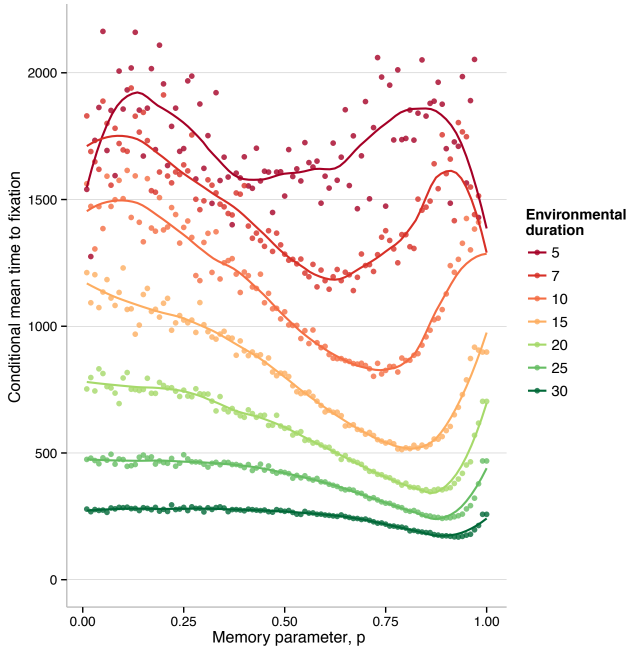

Figure S5: **Conditional mean time to fixation in changing environments.** The y-axis shows conditional mean fixation time for the simulated data in **Figure 3, Panel A**. The curves represent a fit to the data using a generalized additive model with penalized cubic regression splines.

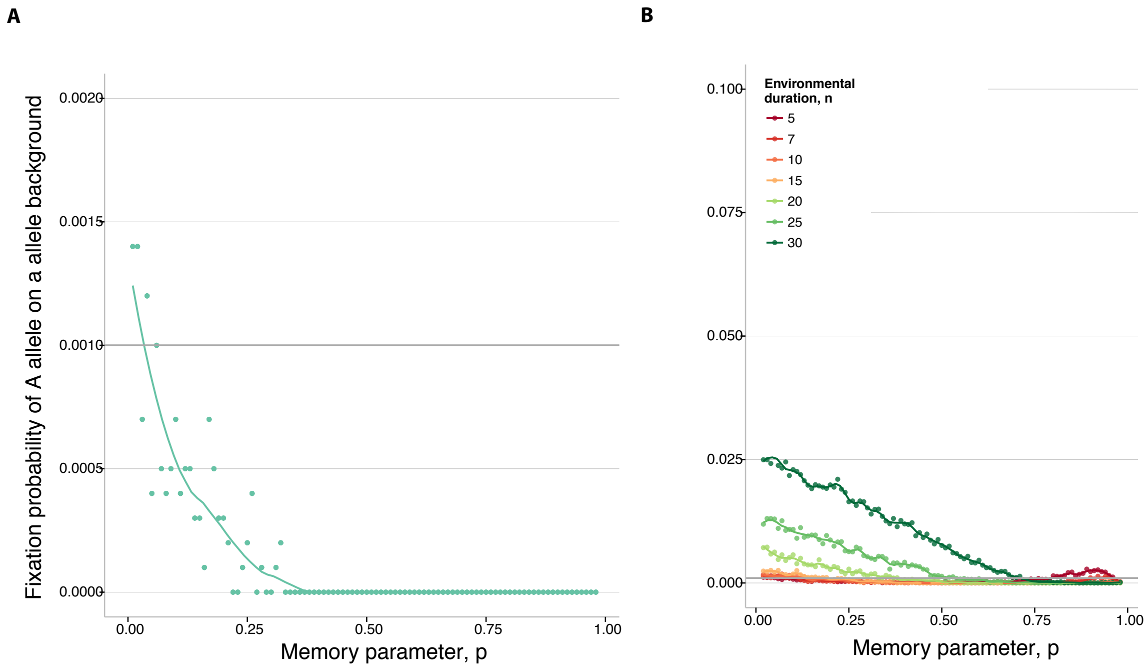

Figure S6: **Counter-fixation probabilities for A allele in a population fixed on a. Panel A:** Constant environment. Parameters are the same as in **Figure 2**. The fixed-phenotype A allele is introduced in a population of a alleles at phenotypic stationary distribution. **Panel B:** Changing environments. Parameters are the same as in **Figure 3, Panel A**. The fixed-phenotype A allele is introduced in a population of a alleles at phenotypic stationary distribution.

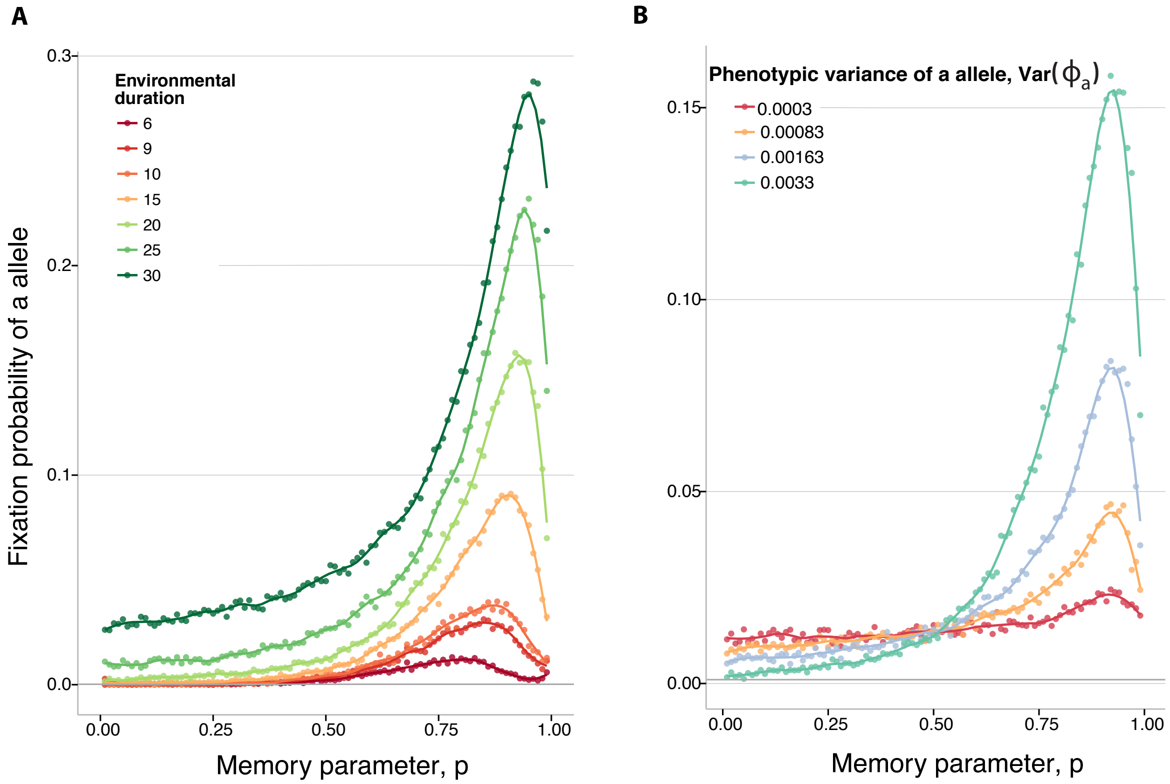

Figure S7: **The phenotypic switching model.** $\mathbb{E}(f^1(\Phi_A)) = 0.6$ and $\mathbb{E}(f^2(\Phi_A)) = 0.8$. The initial environment is E_1. Population size $N = 5000$. **Panel A**: The colors represent different rates of environmental change, n, as presented in the legend. Fitness of a allele sampled between two different phenotypes such that the binomial variance is adjusted to be equal to the equivalent uniform continuous distribution presented in **Figure 2**. **Panel B**: The duration of one environmental stretch is equal to 20 generations. The colors represent different binomial variances of the a allele, $\mathrm{Var}(\Phi_a)$, as presented in the legend. The curves represent a fit to the data using a generalized additive model with penalized cubic regression splines. The first environment is always E_1, in this figure.

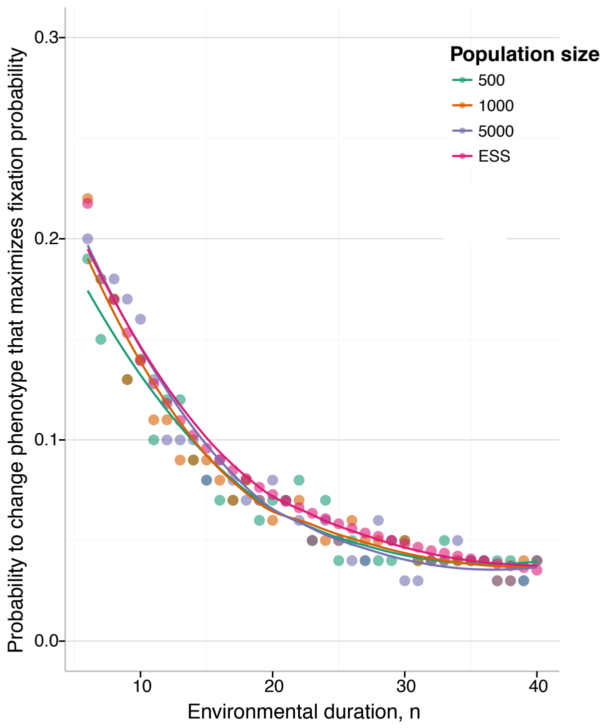

Figure S8: **Phenotypic memory that maximizes the fixation probability with phenotypic switching.** The y-axis shows the phenotypic memory that maximizes the probability of fixation, for different population sizes N. Variance of the a phenotype is $\mathrm{Var}(\Phi_a) = 0.0133$. The ESS switching rates found in the infinite population case are also plotted. The curves represent a fit to the data using a generalized additive model with penalized cubic regression splines.

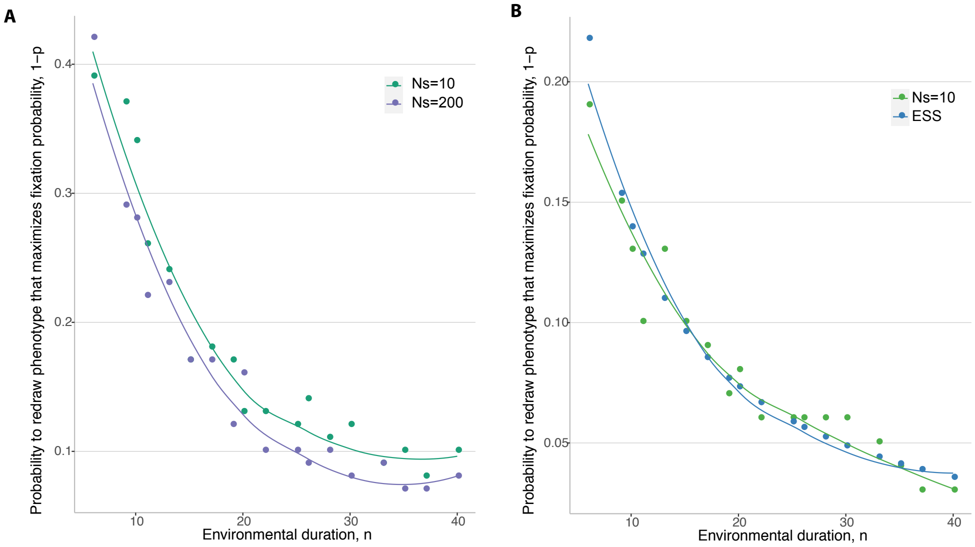

Figure S9: **Phenotypic memory that maximizes the fixation probability for different strengths of selection.** The y-axis shows the phenotypic memory that maximizes the probability of fixation, for different strengths of selection. **Panel A** compares the phenotypic memory that maximizes the fixation probability for both very strong ($Ns = 100$) and moderate to weaker selection ($Ns = 10$), for the continuous phenotype model. Here s denotes the difference in the mean fitness between the two environmental regimes for the two alleles. **Panel B** displays the phenotypic switching rate that maximizes the fixation probability for the two-phenotype switching model and compares it with the ESS switching rate found in the infinite population case. The curves represent a fit to the data using a generalized additive model with penalized cubic regression splines.
